# How I manage differential gas exchange in peripheral venoarterial extracorporeal membrane oxygenation

**DOI:** 10.1186/s13054-023-04703-3

**Published:** 2023-10-27

**Authors:** Richa Asija, Justin A. Fried, Eric C. Siddall, Dana A. Mullin, Cara L. Agerstrand, Daniel Brodie, Joshua R. Sonett, Philippe H. Lemaitre, Darryl Abrams

**Affiliations:** 1 Division of Thoracic Surgery, Columbia University Irving Medical Center, New York, NY USA; 2 Department of Surgery, Community Memorial Health Systems, Ventura, CA USA; 3 Division of Cardiology, Department of Medicine, Columbia University Irving Medical Center, New York, NY USA; 4 Division of Nephrology, Department of Medicine, Columbia University Irving Medical Center, New York, NY USA; 5 Clinical Perfusion, Columbia University Irving Medical Center, New York, NY USA; 6 Division of Pulmonary, Allergy, and Critical Care Medicine, Department of Medicine, Columbia University Irving Medical Center, 177 Ft. Washington Avenue, New York, NY 10032 USA; 7 Division of Pulmonary and Critical Care Medicine, Department of Medicine, Johns Hopkins University School of Medicine, Baltimore, MD USA

**Keywords:** Competitive flow, Dual circulation, Differential oxygenation, Differential carbon dioxide, Mixing point, Venoarterial extracorporeal membrane oxygenation

## Abstract

Dual circulation is a common but underrecognized physiological occurrence associated with peripheral venoarterial extracorporeal membrane oxygenation (ECMO). Competitive flow will develop between blood ejected from the heart and blood travelling retrograde within the aorta from the ECMO reinfusion cannula. The intersection of these two competitive flows is referred to as the “mixing point”. The location of this mixing point, which depends upon the relative strengths of the native and extracorporeal pumps, will determine which regions of the body are perfused with blood ejected from the left ventricle and which regions are perfused by reinfused blood from the ECMO circuit, effectively establishing dual circulations. Because gas exchange within these circulations is dictated by the native lungs and membrane lung, respectively, oxygenation and carbon dioxide removal may differ between regions—depending on how well gas exchange is preserved within each circulation—potentially leading to differential oxygenation or differential carbon dioxide, each of which may have important clinical implications. In this perspective, we address the identification and management of dual circulation and differential gas exchange through various clinical scenarios of venoarterial ECMO. Recognition of dual circulation, proper monitoring for differential gas exchange, and understanding the various strategies to resolve differential oxygenation and carbon dioxide may allow for more optimal patient management and improved clinical outcomes.

## Introduction

Venoarterial extracorporeal membrane oxygenation (ECMO) is a form of mechanical circulatory support used in patients with cardiogenic shock. A peripheral cannulation strategy is commonly employed, typically consisting of a drainage cannula placed in a peripheral vein (e.g. femoral vein), and a reinfusion cannula placed in a peripheral artery (e.g. femoral artery). The management of venoarterial ECMO includes adjusting the blood flow rate to provide sufficient circulatory and oxygenation support and adjusting the sweep gas flow rate to maintain adequate carbon dioxide (CO_2_) removal. A common, but often unrecognized, physiologic phenomenon associated with peripheral venoarterial ECMO is the establishment of dual circulations. The consequences of this phenomenon may be inadequate oxygenation or carbon dioxide removal in certain regions of the body. This phenomenon is most notable with, but not exclusive to, femoro-femoral venoarterial ECMO. Recognition and proper management are critical to optimizing patient outcomes.

### Mechanism of dual circulation

**Fig. 1:**
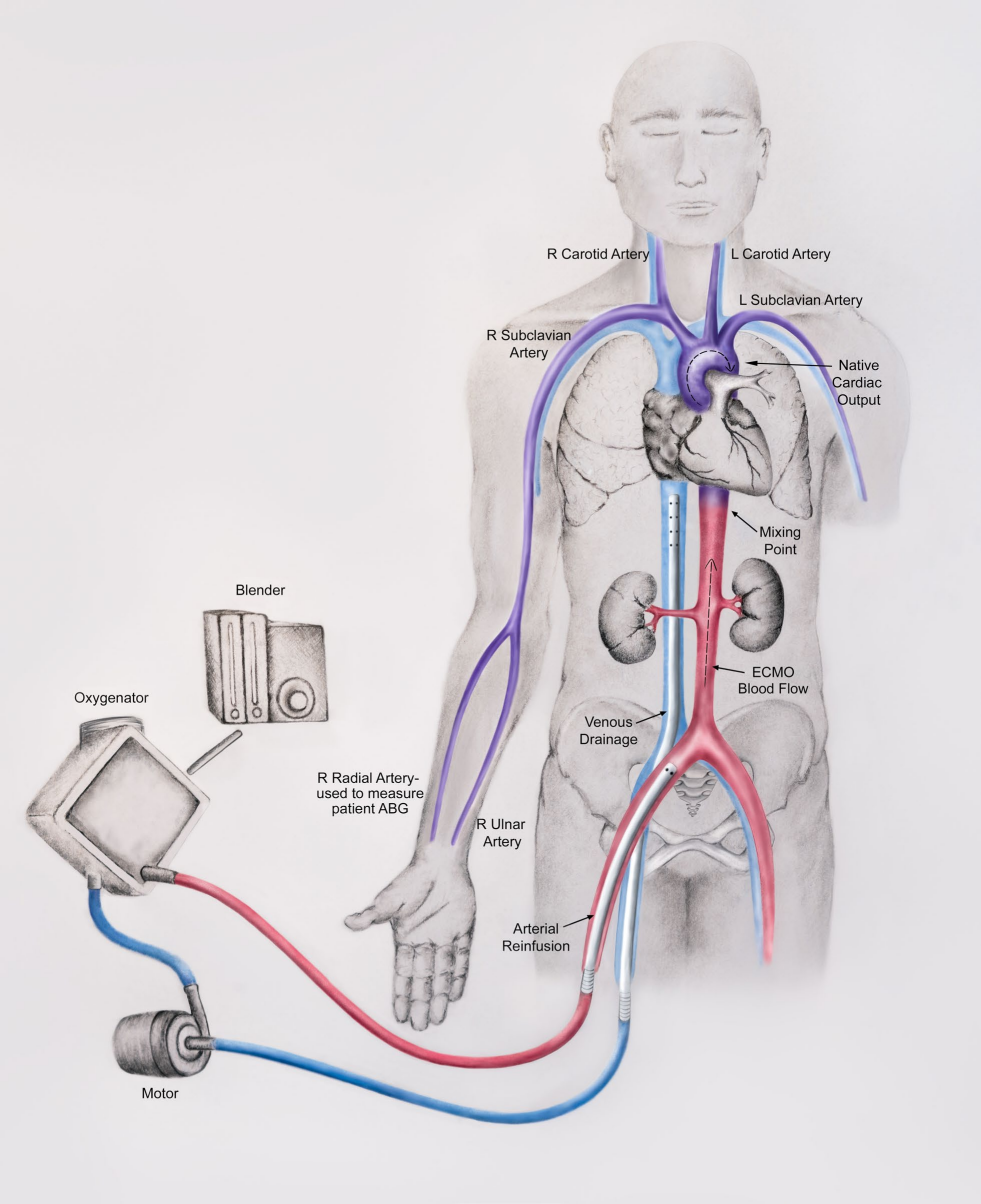
Peripheral femoro-femoral venoarterial extracorporeal membrane oxygenation with differential gas exchange. Red blood: reinfused oxygenated blood from the ECMO circuit. Blue blood: venous blood drained by ECMO circuit. Purple blood: relatively deoxygenated blood ejected from the left ventricle in the setting of impaired native lung gas exchange

Dual circulation is a phenomenon seen with venoarterial ECMO in which different anatomic portions of the body are perfused by either native cardiac output or reinfused ECMO blood flow. As long as the heart ejects, competitive flow will develop with peripheral venoarterial ECMO cannulation strategies between blood ejected from the heart and blood travelling retrograde within the aorta from the ECMO cannula. The intersection of these two competitive flows is referred to as the “mixing point”. The location of this mixing point will depend on the relative strengths of the native and extracorporeal pumps and will determine which areas of the body are perfused with blood ejected from the left ventricle and which areas of the body are perfused by the reinfused blood from the ECMO circuit [1, 2]. In the absence of native cardiac output (e.g. cardiac arrest), there is neither a mixing point nor dual circulation, as all aortic perfusion comes from the ECMO cannula. In severe cardiogenic shock, with minimal native cardiac output, the mixing point may be located near the aortic root or in the ascending aorta. With increasing amounts of cardiac output, the mixing point moves progressively more distal in the aorta. The mixing point can also change as ECMO flow is altered; higher ECMO blood flow rates move the mixing point more proximally within the aorta while a decrease in ECMO blood flow rate moves the mixing point more distally. In configurations where the reinfusion cannula is closer to the aortic root (e.g. subclavian artery, innominate artery or ascending aorta), more of the ascending aorta and aortic arch will be supplied by extracorporeal blood flow, and dual circulation will be less pronounced. This anatomical consideration is critical since the coronary circulation arises from the aortic root and cerebral circulation arises from the aortic arch (Fig. 1).

### Impact of dual circulation on gas exchange in different regions of the body

The oxygen and carbon dioxide content of blood ejected by the native cardiac output is determined by gas exchange within the native lung, whereas oxygen and carbon dioxide content in reinfused ECMO blood is determined by gas exchange within the membrane lung of the ECMO circuit. When there is native left ventricular output with concomitant impaired native gas exchange, poorly oxygenated blood ejected from the left ventricle will be delivered to the vascular beds proximal to the mixing point, whereas the systemic vasculature distal to the mixing point will be perfused by well-oxygenated reinfused blood from the ECMO circuit, a phenomenon referred to as “differential oxygenation [3]”. If the mixing point was to occur in the descending thoracic aorta, for example, the poorly oxygenated blood from the native cardiac circulation would perfuse the upper body (including the carotid and coronary arterial systems), whereas well-oxygenated blood reinfused from the ECMO circuit would perfuse the lower body (including the renal and mesenteric arteries).

In order to estimate the quality of upper body (carotid and cerebral) oxygenation, oxygen saturation of the right upper extremity (measured by either pulse oximetry or arterial blood gas (ABG) analysis) can be used as a surrogate to reflect oxygen delivery to the innominate artery and thus the cerebral and carotid vessels. Well-oxygenated blood from the right arm of a patient with impaired native lung gas exchange suggests that the aortic arch is being perfused by reinfused blood from the ECMO circuit and that the carotid arteries and cerebral vasculature are adequately oxygenated (though with uncertainty about oxygenation of the coronary arteries). Poorly oxygenated blood from the right arm of this same patient indicates that the mixing point is distal to the innominate artery—making it less certain that the carotid arteries are adequately oxygenated and likely that the coronary vasculature is poorly oxygenated.

A less well-recognized phenomenon, though conceptually similar to differential oxygenation, is differential carbon dioxide. In such circumstances, carbon dioxide content proximal to the mixing point is determined by native lung ventilation and carbon dioxide content distal to the mixing point is determined by the sweep gas flow rate of the ECMO circuit, with the potential for hypercapnic blood being delivered to one region of the body and normocapnic or hypocapnic blood delivered to a different region, depending on the relative rates of carbon dioxide removal of the native and membrane lungs.

In this review, we will discuss different real-world scenarios of venoarterial ECMO that exemplify the issues encountered with dual circulation, the resultant differential oxygenation and carbon dioxide, and how we recommend managing these patients.

### Scenario 1: lack of competitive flow, no dual circulation

A 67-year-old woman experienced a ventricular fibrillation arrest refractory to cardiopulmonary resuscitation and defibrillation. She was emergently cannulated to femoral venoarterial ECMO. Immediately after ECMO cannulation, she was noted to have similar partial pressures of oxygen and carbon dioxide in her post-membrane lung ABG and her right radial ABG. This demonstrated that due to severe myocardial dysfunction after cardiac arrest, the mixing point between native and extracorporeal blood flows was proximal to the take-off of the innominate artery from the aortic arch and the oxygenated blood from the ECMO circuit was perfusing the systemic vasculature of her upper and lower body. As the heart likely had minimal left ventricular function after the arrest, there was nominal competitive flow from native cardiac function against the ECMO reinfusion flow. With no evidence of significant dual circulation in this scenario, changes in either the sweep gas flow rate or the fraction of oxygen delivered by the ECMO circuit would affect both upper and lower body gas exchange equally.

### Scenario 2: dual circulation with impaired native gas exchange

A 48-year-old woman with interstitial lung disease and pulmonary hypertension was admitted to the intensive care unit for acute on chronic hypoxemic respiratory failure and right ventricular failure, with preserved left ventricular systolic function. She was placed on peripheral venoarterial ECMO after exhibiting signs of worsening right ventricular function. She received intravenous fluids to maintain adequate ECMO blood flow, but this led to pulmonary oedema with subsequent worsening of upper body oxygenation as measured on her right radial ABG, which showed a pH of 7.36, partial pressure of carbon dioxide (PaCO_2_) of 53 mm Hg and partial pressure of oxygen (PaO_2_) of 66 mm Hg. She had multiple desaturation events with worsening shortness of breath despite maximal non-invasive supplemental oxygen, the addition of inhaled nitric oxide, and increases in ECMO blood flow rate. Her preserved left ventricular function combined with impaired native lung gas exchange resulted in the delivery of hypoxemic blood to the innominate artery and potentially to additional vessels off the aortic arch, depending on the precise location of the mixing point. Oxygenation, as supplied by the ECMO circuit, was limited to the vasculature distal to the mixing point.

In order to improve gas exchange to the upper body in this situation, the decision was made to reconfigure her ECMO circuit to venoarterial–venous (VAV) ECMO. An additional reinfusion cannula was placed in the right internal jugular vein and spliced to the reinfusion line using a Y connector, allowing well-oxygenated blood to be reinfused into a central vein, which would then be propagated through the native cardiac circulation and ejected into the ascending aorta. This hybrid approach—arterial reinfusion for circulatory support combined with venous reinfusion for upper body gas exchange support—can be performed percutaneously at the bedside and can therefore be instituted as soon as it is evident that femoral venoarterial ECMO cannulation alone is inadequate to support upper body gas exchange [4]. How one chooses to split the reinfused ECMO blood flow in this configuration—which may be controlled by using a clamp to create resistance on the venous reinfusion limb—will be dictated by how much blood flow is needed in the venous limb to adequately support upper body gas exchange and how much blood flow is needed in the arterial limb for circulatory support. To minimize the risk of thrombus formation in each limb, a minimum ECMO blood flow is recommended in each limb (e.g. 1–1.5 L per minute). An alternative strategy could have been to convert her from femoral venoarterial ECMO to an upper body venoarterial configuration (e.g. internal jugular venous drainage and reinfusion to the axillary, subclavian, or innominate artery via an end-to-side graft) or directly into the ascending aorta, all of which would reduce the extent of dual circulation and differential oxygenation, as the mixing point would be increasingly closer to the aortic root. These approaches, however, are more invasive and cannot be performed at the bedside.

### Scenario 3: dual circulation with preserved native gas exchange but inadequate ECMO sweep gas flow rate

A 56-year-old woman recently diagnosed with pulmonary hypertension secondary to end-stage interstitial lung disease presented with decompensated pulmonary hypertension and right ventricular failure, with associated high vasopressor requirements, renal failure, and lactic acidosis. She was placed on femoral venoarterial ECMO with marked improvement in her shock, renal function, and lactate. Her initial right radial ABG showed a pH of 7.36, PaCO_2_ of 41 mm Hg and PaO_2_ of 194 mm Hg, which is more reflective of preserved native gas exchange (with an appropriate response to supplemental oxygen) rather than reinfused ECMO blood flow with a post-membrane gas showing pH of 7.33, PaCO_2_ of 47 mm Hg and PaO_2_ of 562 mm Hg. Over the next three days, however, she developed a primary metabolic alkalosis (based on ABGs and chemistry panels drawn from the right radial artery). This acid–base derangement was initially attributed to a contraction alkalosis in the setting of diuresis. Her right radial ABG showed a pH of 7.59 mm Hg, PaCO_2_ of 35 mm Hg and bicarbonate of 33 mmol/L consistent with a mixed metabolic alkalosis and respiratory alkalosis, whereas a post-membrane blood gas, representing blood reinfused to the lower body through the ECMO circuit, showed a pH of 7.41, PaCO_2_ of 55 mm Hg and a bicarbonate of 33 mmol/L. Diuretics were held and she received potassium chloride without improvement in the metabolic alkalosis. In an attempt to correct the upper body alkalemia, the sweep gas flow rate was decreased to reduce CO_2_ removal.

Over the subsequent days, her upper body metabolic alkalosis worsened (right radial ABG: pH 7.53, PaCO_2_ 47 mm Hg, and bicarbonate 39 mmol/L), though the pH was improved owing to appropriate respiratory compensation. At the same time, she had a primary respiratory acidosis in the lower body with appropriate renal compensation (post-membrane blood gas: pH 7.32, PaCO_2_ 97 mm Hg and bicarbonate 51 mmol/L). The changes in the right radial and post-membrane blood gases suggest that the decrease in sweep gas flow rate led to worsening respiratory acidosis in the blood delivered to the kidneys, whose response was to increase hydrogen ion excretion (increase generation of bicarbonate) ultimately leading to a worsening systemic metabolic alkalosis (including in the upper body). During this time, the brain, sensing a metabolic alkalosis, demonstrated appropriate respiratory compensation by reducing native lung ventilation, but such a change in CO_2_ was isolated to the blood proximal to the mixing point (i.e. the upper body).

In this patient with preserved native gas exchange, the assumption was that minimal sweep gas would be needed to manage CO_2_. However, in the presence of dual circulation with a mixing point superior to the renal arteries, the carbon dioxide tension of blood within the lower body was dependent on the sweep gas flow rate of the ECMO circuit, rather than native lung ventilation. The pH of the blood perfusing the kidneys determines the renal response to an acid–base disturbance. At the same time, the pH of the blood perfusing the brain determines the ventilatory response to an acid–base disturbance. Given that there were differential pH values in the blood perfusing the brain and kidneys, the compensatory responses were independent of each other. A low sweep gas flow resulted in the delivery of acidemic blood with high CO_2_ to the kidneys which responded appropriately by increasing hydrogen ion excretion (thus raising the serum bicarbonate). The increase in serum bicarbonate was commensurate with the pH perfusing the kidneys as driven by the ECMO circuit. Since the upper body was alkalemic to start with, an increase in serum bicarbonate produced by the kidneys exacerbated upper body metabolic alkalosis (even though pH improved since respiratory compensation was appropriate). In a counterintuitive way, to improve the upper body alkalemia, we then increased the sweep to remove more CO_2_ in the lower body which prompted bicarbonate excretion by the kidneys. The eventual pH normalization in the upper body confirmed that this was both the correct mechanism and the appropriate corrective action.

This table is a summary of the scenarios and the recommended management. Of note, this does not include pH changes from separate metabolic processes (lactic acidosis, renal failure, etc.) (Table 1).

**Table 1:** Summary of venoarterial ECMO scenarios with recommended management

	Scenario 1	Scenario 2	Scenario 3
Upper body Oxygenation	High	Low	Normal
Upper body PaCO_2_	Normal^1^	Normal/high^3^	Normal/high^4^
Upper body pH	Normal^2^	Normal/low^3^	High
Lower body Oxygenation	High	High	High
Lower body PaCO_2_	Normal^1^	Normal^1^	High
Lower Body pH	Normal^2^	Normal^1^	Low
Upper/lower body pH ratio	1	< 1	> 1
Management	Adjust sweep as needed to maintain adequate upper body pH and PaCO_2_	ECMO circuit reconfiguration to VAV ECMO or upper body VA ECMO	Increase sweep, creating lower body alkalemia in order to prompt the kidneys to eliminate bicarbonate

*ECMO* extracorporeal membrane oxygenation,* PaCO*_*2*_ partial pressure of carbon dioxide in arterial blood,* VA* venoarterial,* VAV* venoarterial venous

^1^Assuming sweep is adjusted to a normal pH

^2^In the absence of an independent metabolic acidosis or alkalosis

^3^Depending on how well preserved or impaired native lung ventilation may be

^4^Upper body PaCO_2_ may be high as a compensatory response to the metabolic alkalosis

## Conclusion

Understanding the concept of dual circulation in venoarterial ECMO and the various ways it can manifest is critical in the management of this phenomenon. Recognition of differing levels of oxygen and carbon dioxide in different regions of the body will help inform the appropriate management strategy, which, depending on the circumstances, may require adjustments in sweep gas flow rates or reconfiguration of the ECMO circuit.

## Data Availability

Not applicable.

## Abbreviations

ABG: Arterial blood gas
ECMO: Extracorporeal membrane oxygenation
PaCO_2_: Partial pressure of carbon dioxide
PaO_2_: Partial pressure of oxygen
VAV: Venoarterial–venous
